# Targeting Mitochondrial Oncometabolites: A New Approach to Overcome Drug Resistance in Cancer

**DOI:** 10.3390/pharmaceutics13050762

**Published:** 2021-05-20

**Authors:** Martina Godel, Giacomo Ortone, Dario Pasquale Anobile, Martina Pasino, Giulio Randazzo, Chiara Riganti, Joanna Kopecka

**Affiliations:** Department of Oncology, University of Torino, via Santena 5/bis, 10126 Torino, Italy; martina.godel@edu.unito.it (M.G.); giacomo.ortone@edu.unito.it (G.O.); dario.anobile@edu.unito.it (D.P.A.); martina.pasino530@edu.unito.it (M.P.); giulio.randazzo754@edu.unito.it (G.R.); chiara.riganti@unito.it (C.R.)

**Keywords:** mitochondrial oncometabolites, cancer drug resistance, cancer metabolism

## Abstract

Drug resistance is the main obstacle for a successful cancer therapy. There are many mechanisms by which cancers avoid drug-mediated death, including alterations in cellular metabolism and apoptotic programs. Mitochondria represent the cell’s powerhouse and the connection between carbohydrate, lipid and proteins metabolism, as well as crucial controllers of apoptosis, playing an important role not only in tumor growth and progression, but also in drug response. Alterations in tricarboxylic acid cycle (TCA) caused by mutations in three TCA enzymes—isocitrate dehydrogenase, succinate dehydrogenase and fumarate hydratase—lead to the accumulation of 2-hydroxyglutarate, succinate and fumarate respectively, collectively known as oncometabolites. Oncometabolites have pleiotropic effects on cancer biology. For instance, they generate a pseudohypoxic phenotype and induce epigenetic changes, two factors that may promote cancer drug resistance leading to disease progression and poor therapy outcome. This review sums up the most recent findings about the role of TCA-derived oncometabolites in cancer aggressiveness and drug resistance, highlighting possible pharmacological strategies targeting oncometabolites production in order to improve the efficacy of cancer treatment.

## 1. Introduction: Mitochondria in Cancer

Treating advanced tumors is still an important challenge because of the concomitant presence of intrinsic and acquired resistance to the commonly used anti-cancer drugs. Most advanced tumors share the ability to escape cell death mediated by anticancer drugs, while continuing their growth and progression. The main mechanisms responsible for drug resistance, often known as multidrug resistance since it involves multiple drugs with different mechanisms of action, are the reduced drug uptake and accumulation, the increased drug efflux via membrane transporters that decrease intratumor drug concentration and cytotoxicity, the efficient mechanisms of DNA repair, the bypass of the DNA damaging and the cell cycle arrest induced by many chemotherapeutics, the prevalence of survival pathways over apoptotic pathways, the metabolic reprograming [1]. Inhibition of apoptosis and metabolic rewiring are strongly correlated with altered mitochondria functions, protect cancer cells from drug-mediated death inducing drug resistance [2].

Traditionally, the shift from oxidative phosphorylation (OXPHOS) to aerobic glycolysis is considered a hallmark of cancer, postulated by Otto Von Warburg. This implies that mitochondria are poorly active in tumors [3]. However, an increasing amount of evidence demonstrates that, despite several cancers satisfying their energetic requirements using glycolysis, other tumors—in particular if drug resistant—heavily rely on OXPHOS to fuel their metabolism [4].

For many years, mitochondria have been considered only as the energy powerhouse of the cell. Nonetheless, in addition to their role in generating ATP, mitochondria are key signaling centers regulating cancer development and progression, including metabolic reprogramming in response to anticancer drugs. In fact, the mitochondrial oxidative decarboxylation of pyruvate, the fatty acid β-oxidation (FAO) and the glutaminolysis are the key fuel of the tricarboxylic acid (TCA) cycle that sustains production of ATP via OXPHOS. The mitochondrial intermediate metabolism interconnects amino acid, lipid and carbohydrate metabolism that are involved in both TCA anaplerotic fluxes, to obtain energy equivalents, and TCA cataplerotic fluxes towards the synthesis of building blocks such as proteins and nucleotides. Higher energetic mitochondrial metabolism, higher anaplerotic and cataplerotic fluxes are hallmarks of drug resistant cancers [5]. Indeed, this metabolic phenotype replenishes cancer cells either of ATP and building blocks, attenuating the damages of cytotoxic stresses, including chemotherapy. Moreover, blocking the pyruvate dehydrogenase complex, i.e., the influx of glucose-derived acetyl-CoA into TCA cycle and/or anaplerotic pathways (such as FAO, glutaminolysis, glutamate oxidative metabolism, arginine, proline, asparagine, aspartate and phenylalanine catabolism) [6], buffers production of the reactive oxide species (ROS) by electron transport chain (ETC) [7], maintaining them below a “danger threshold” and determining the production of low levels of ROS that train cancer cells to resistance to oxidative stress and chemotherapeutic drugs, by upregulating antioxidant mechanisms [8].

Beside a key role in energetic metabolism and ROS production, mitochondria regulate the apoptotic response upon chemotherapy, by decreasing the BCL2/BAX ratio, increasing the permeabilization of mitochondrial membrane, the opening of mitochondrial permeability transition pore and the release of cytochrome c, which activates the apoptosome and the caspase 9/3 axis [9]. Furthermore, mitochondrial DNA (mtDNA) is frequently mutated in tumors, spontaneously or upon the damage induced by chemotherapeutic drugs, such as cisplatin and gemcitabine [10]. Since mtDNA mainly encodes for mitochondrial translation machinery and ETC complexes, mutations in these key players of OXPHOS may produce the synthesis of complexes characterized by a defective reduction of electron shuttles (ubiquinone, cytochrome c), structural components (Fe-S cluster-, cytochrome-containing proteins) or O_2_, determining the generation of radical species or ROS. This mitochondrial dysfunction promotes metabolic alterations, changes the ROS buffering and the balance between pro-apoptotic and anti-apoptotic signaling, contributing to drug resistance [11].

Genetic alterations of TCA cycle enzymes such as succinate dehydrogenase (SDH), also shared with complex II of ETC, fumarate hydratase (FH) or isocitrate dehydrogenase (IDH) lead to the accumulation of the upstream intermediates—succinate, fumarate and 2-hydroxyglutarate (2-HG), respectively. They are known as oncometabolites, because of their role in cancer growth, aggressiveness and progression [12]. This review will describe the current understanding of oncometabolite role in cancer aggressiveness, with a special emphasis on drug resistance. Next, we will focus on potential pharmacological strategies targeting the production of oncometabolites as potential tools improving the efficacy of anti-cancer treatments.

## 2. Mitochondrial Oncometabolites and Cancer Biology

Succinate, fumarate and 2-HG dysregulate a plethora of cellular processes associated with invasiveness and drug resistance, such as protein post-translational modifications, metabolic and epigenetic events, epithelial-to-mesenchymal transition (EMT), inhibition of α-ketoglutarate (α-KG)-dependent dioxygenase enzymes [12]. Since α-KG is part of TCA cycle, its modulation by the oncometabolites generated within the TCA cycle itself may represent a finely tuned feedback control on the cycle itself.

Succinate is generated from succinyl-CoA and oxidated into fumarate by SDH. Fumarate is the substrate for FH, an enzyme that catalyzes the reversible hydration/dehydration of fumarate to malate. SDH complex is built of four subunits (SDHA, SDHB, SDHC, SDHD). It is the only TCA enzyme that produces FADH2 [12,13]. Mutations in SDH have been reported in all subunits. In addition, some SDH-deficient tumors either have hypermethylation in the SDHC promoter, which phenotypically makes these tumors without a functioning SDH [14] or have hyper-expressed the tumor necrosis factor-associated protein (TRAP1) that inhibits SDH [15]. Moreover, down-regulation of SDH mRNA by miR-210, miR-31 and miR-37, post-translational modifications such as dephosphorylation by PTEN-like mitochondrial phosphatase-1, deacetylation of lysines consequent to the loss of sirtuin 3, competitive inhibition by itaconate, a TCA cycle side-metabolite produced by the decarboxylation of cis-aconitate during inflammation [16], result in the inhibition of SDH activity [17]. In all these cases succinate accumulates, as reported in paragangliomas, pheochromocytomas, neuroblastomas [18], gastrointestinal stromal cancer [19], colon [20], renal and ovarian cancers [21].

FH has isoforms with different cellular localization: one mitochondrial isoform participates in TCA cycle; one cytosolic isoform is involved in the metabolism of amino acids and fumarate [22]. When FH is mutated, as described for instance in renal cell cancer (RCC) and kidney renal papillary cell carcinoma (KIRP) [23], fumarate accumulates [13]. Importantly, RCC with mutant FH is one of the most aggressive forms of renal cancer, characterized by early metastasis and a poor clinical outcome [24]. Sporadic cases of deficient FH were also reported for paragangliomas, pheochromocytomas [25], neuroblastoma [26], uterine and skin leiomyoma [27], and endometrial cancer [28]. A similar metabolic phenotype with fumarate accumulation is described in nasopharyngeal carcinomas overexpressing the lymphoid-specific helicase, a chromatin remodeling ATPase that causes FH repression: this phenotype has been linked to increased migration and invasion [29].

Differently from succinate and fumarate, 2-HG is produced by an abnormal catalytic activity of IDH, a TCA enzyme that in physiological conditions catalyzes the decarboxylation of isocitrate into α-KG and CO_2_, using NADP+ and Mg^2+^ as cofactors [30]. The reverse reaction produces isocitrate through the reductive carboxylation of α-KG, consuming NAD(P)H and CO_2_: when this reaction is incomplete and does not involve CO_2_, α-KG is reduced into 2-HG [31]. IDH enzymes are present in three isoforms, each with a different subcellular localization and co-substrates specificity: IDH1 is localized in peroxisomes and cytosol and uses NADP+/NADPH, the NADP+/NADPH-dependent IDH2 and the NAD+/NADH-dependent IDH3 are present in mitochondria. IDH1 reversibly interconverts α-KG into isocitrate as part of the reductive glutamine metabolism [32]. IDH2 is mainly involved in the oxidative metabolism of isocitrate in the TCA cycle, while IDH3 irreversibly oxidizes isocitrate to produce α-KG and NADH. Somatic point mutations in IDH1 and IDH2 genes have been found in glioma, glioblastoma multiforme (GBM) and acute myeloid leukemia (AML), prevalently concentrated in specific hot spots [30]. The IDH1 R132H/C/Q, IDH2 R140Q/W/L and R172K/M/G/T/S are the most common mutations conferring a new catalytic activity with overproduction of 2-HG [33].

Succinate, fumarate and 2-HG contribute to cancer growth with pleiotropic mechanisms, such as stabilization of the hypoxic inducible factor-1α (HIF-1α), epigenetic changes, apoptosis alteration, increased production of mitochondrial ROS (mtROS) and protein or chromatin “succinylation”, all events that occur often concurrently and are interconnected [34].

The accumulation of succinate in tumors causes the so-called pseudohypoxia, a condition characterized by the stabilization of HIF-1α notwithstanding the normoxic environment. This pseudohypoxic phenotype promotes cell survival, proliferation, angiogenesis and drug resistance [35]. Succinate mediates HIF-1α stabilization by inhibiting the prolyl-hydroxylases (PHDs), which are responsible for hydroxylating HIF-1α and marking it for proteasomal degradation. The mechanism of PHDs inhibition by succinate was first demonstrated in HEK293 cells silenced for SDHD subunit that has increased succinate and lacked HIF-1α degradation [36]. The same results were obtained in the human colon cancer cell line HCT116, knocked-down for SDHB, where succinate accumulation and HIF-1α stabilization were accompanied by lower mitochondrial O_2_ consumption rate and higher extracellular acidification, indicative of a metabolic shift toward glycolysis induced by HIF-1α [37]. Succinate may act also through succinate receptor SUCNR1, which stabilizes HIF-1α in non-small cell lung cancer by engaging the downstream mediators phosphatidylinositol 3-phosphate kinase (PI3K)/Akt that phosphorylate HIF-1α on serine [38]. Of note, SUCNR1 is highly expressed also in kidney cancer, where its signaling promotes angiogenesis, hematopoiesis and inflammation [39], and in tumor-associated macrophages (TAMs), where the binding of succinate favors the polarization toward a tumor-permissive M2-phenotype, facilitating cancer cell migration, invasion and metastasis [38]. Further, the increase in mtROS that is associated with SDH deficiency [40] may increase HIF-1α because ROS inactivate PHDs, thus preventing HIF-1α degradation [41,42]. In neuroblastoma, the increase of HIF-1α has also been caused by the inhibition of the ten-eleven translocation proteins (TETs) that antagonize DNA methylation in specific loci by oxidizing 5-methylcytosines. Both succinate and fumarate inhibited TETs and induced the simultaneous transcriptional increase of HIF-1α and HIF-2α. However not all hypoxia-responsive genes were upregulated [26], suggesting that differential circuitries—dependent and independent from mitochondrial oncometabolites—control the transcriptional activity of HIFs. In addition, 2-HG causes pseudohypoxia: in the presence of mutated IDH2, the high ratio between 2-HG and α-KG reduces the activity of PHDs, stabilizing HIF-1α [33].

As mentioned before, mitochondrial oncometabolites cause epigenetic changes in cancer cells, boosting oncogenesis and cancer progression. SDH deficient tumors are characterized by hypermethylation of histones and DNA cytosine [14,43], as a result of the succinate-mediated inhibition of histone lysine demethylases (KDMs) and TETs [26,44]. The hypermethylation changes the expression profile of specific genes, leading for instance to the activation of the EMT program, as demonstrated in pheochromocytomas and paragangliomas knocked-down for SDHB [45]. Moreover, fumarate inhibits TETs and KDMs, suppressing the anti-metastatic miR-200, up-regulating specific transcription factors, such as Twist and HIF-1α, that promote EMT. As proof of concept, the reintroduction of full-length Fh1 in mouse and human FH-deficient cells was sufficient to prevent the EMT signature, reducing vimentin and restoring E-cadherin expression [46]. In addition, epigenetic changes in FH deficient cells can contribute to defects in DNA Damage Response (DDR) and to the bypass of the cell cycle checkpoints activated after DNA damage, e.g., after irradiation. In vitro studies on RCC-derived cell lines showed that FH deficiency causes either the arrest in G1-phase or the more rapidly progress through mitosis after DNA damage. Fumarate accumulation enhances the bypass of G2-phase checkpoints, activates the error-prone non-homologous end-joining (NHEJ) repair system during cell mitosis, and favors the accumulation of ROS, known inducers of DNA damage, with the result of an increased genome instability [47]. The effects of fumarate in chromatin remodeling and DNA damage are not tumor specific, because the DNA hypermethylation observed in HepG2 cells exposed to millimolar concentrations of fumarate were comparable to the hypermethylation detected in FH deficient cells [48]. Of note, hypermethylation can be also caused by 2-HG that acts at the epigenetic level by competitively inhibiting TET2 and JMJD2A/lysine demethylase 4A [33].

FH plays a physiological role in controlling cell proliferation, independent on the effects on cell cycle and chromatin remodeling. This role is often altered in cancer cells, as a consequence of the activation of other pathways peculiar of transformed cells, such as the O-glycosylation (O-GlcNAc) pathway or the p21 Activated Kinase 4 (PAK4)-dependent activity. While in non-transformed cells, FH binds the Activation Transcription Factor 2 (ATF2) and enhances its transcriptional activity, favoring cell cycle arrest, in cancer cells, particularly in those tumors that display a high activity of O-GlcNAc transferase (OGT) such as pancreatic adenocarcinoma (PDAC) [49], the FH/ATF2-mediated events are impaired by the O-GlcNAc glycosylation of FH. This post-translational modification limits the interaction between FH and ATF2, maintaining high levels of cell proliferation [50]. Accordingly, PDAC patients with high OGT and O-GlcNAc-FH levels have a lower median survival [50]. Transforming growth factor β (TGFβ) is responsible for a second mechanism of fumarate-dependent inhibition of cell proliferation, disrupted in cancer cells. TGFβ signaling favors growth arrest by increasing the phosphorylation of FH on Thr90 by p38 mitogen activated kinase (MAPK). Such phosphorylation favors the interaction between FH and recombination signal binding protein for immunoglobulin kappa J region, also known as CSL, a downstream effector of Notch. The FH/CSL complex associates p53 and is recruited on the promoter of the p53-targeted gene p21/cyclin dependent kinase inhibitor 1A, which prevents cell cycle progression. PAK4, highly expressed in lung cancers, counteracts the anti-proliferative effect induced by TGFβ/FH/CSL cascade by phosphorylating FH on Ser46, an event that—contrarily to the phosphorylation on Thr90—impairs the interaction between FH and CSL, favoring tumor proliferation and metastasis. Conversely, the PAK4 inhibitor PF-3758309 favors the FH-CSL interaction in non-small cell lung cancer cells and enhances the anti-proliferative effect induced by TGFβ [51].

Multiple pathways regulating cell proliferation and/or inhibiting of apoptosis are controlled also by 2-HG [31]. In IDH1-R132Q knock-in mutant cells, 2-HG physically binds Cdc42, a small GTPase of Rho family involved in the regulation of cell cycle. By doing so, 2-HG blocks Cdc42 interaction with mixed lineage kinase 3 (MLK3), a component of the pro-apoptotic cascade MLK3/MKK4-7/JNK/Bim. Typically, 2-HG prevents the association between Cdc42 and MLK3, preventing apoptosis and favoring cancer cells proliferation and tumor mass expansion [52]. Moreover, it has been reported an important decrease of p53 in mouse embryonic fibroblasts and HCT116 cells carrying IDH1-R132Q/R132H mutations: the high levels of 2-HG stabilize HIF-2α, which activates the transcription of miR-380-5p. The latter triggers the degradation of p53 mRNA, favoring cell proliferation and tumorigenesis [30]. In agreement, the levels of p53 are negatively correlated with IDH1-R132H levels in human gliomas [30], supporting at clinical levels the molecular mechanism dependent on the 2-HG/HIF-2α/miR-380-5p axis described in vitro. A p53-independent mechanism by which 2-HG prevents apoptosis has been documented in AML cells engineered to express mutant IDH1-R132H, where 2-HG accumulates and inhibits the cytochrome c oxidase complex of the ETC. This metabolic shut down increases the activation of the anti-apoptotic BCL2 protein, promoting tumor growth and progression [33,53], likely by reducing mtROS.

**Figure 1:**
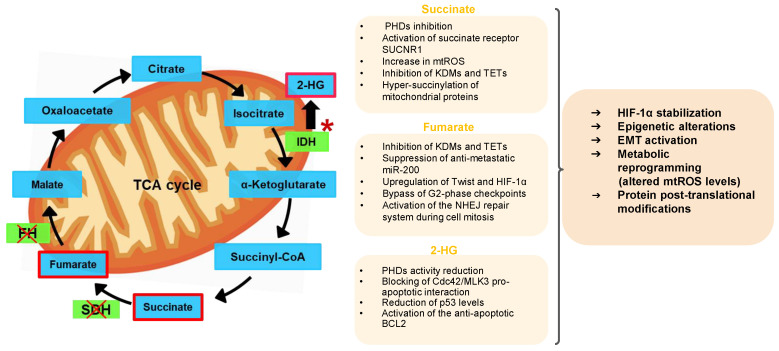
TCA cycle, main oncometabolites and effects on cancer biology. An altered tricarboxylic acid (TCA) cycle due to gain-of-function mutations (*) in isocitrate dehydrogenase (IDH) and loss-of-function mutations (X) in succinate dehydrogenase (SDH) and fumarate hydratase (FH), produces high levels of 2-hydroxyglutarate (2-HG), succinate and fumarate, respectively, that act as oncometabolites by pleiotropic mechanisms. PHDs: prolyl-hydroxylases; SUCNR1: succinate receptor 1; mtROS: mitochondrial reactive oxygen species; KDMs: histone lysine demethylases; TET: ten-eleven translocation proteins; HIF-1α: hypoxia-induced transcriptional factor; NHEJ: non-homologous end-joining; Cdc42: cell division control protein 42; MLK3: mixed lineage kinase 3; BCL2: B-cell lymphoma 2; EMT: epithelial mesenchymal transition.

A peculiar mechanism that links the production of TCA-derived oncometabolites to oncogenesis and tumor progression is proteins succinylation, a post-translational modification in which succinyl group is added to a lysine residue. After the attachment of succinate, the positive charge of lysine is negatively charged, leading to important changes in protein structure and function. Although hyper-succinylated proteins have been already associated with the tumors with SDH, FH and IDH deficiency, the impact of this post-translational modification in cancer cells is still matter of investigation [54]. The best studied protein regulated by succinylation is Kelch-like ECH-associated protein 1 (KEAP1), the endogenous inhibitor of the redox-sensitive transcription factor Nuclear Factor, Erythroid 2 Like 2 (NRF2). KEAP1 succinylation prevents its binding to NRF2, activating the NFR2-mediated transcriptional program, including several genes involved in the antioxidant response [55]. Interestingly, the hyper-succinylation of several mitochondrial proteins in SDH and IDH deficient cells may alter mitochondrial-dependent apoptosis and metabolism. For instance, hyper-succinylated proteins in mitochondria increase the association of the pro-survival BCL-2 protein to the mitochondrial membrane [56], a condition that confers resistance to apoptosis. An intriguing cross-talk occurs between the three enzymes of TCA cycle producing oncometabolites in glioma with mutated IDH1. Since 2-HG is a structural analogue of succinate and fumarate it may inhibit both SDH and FH, determining the concurrent accumulation of succinate and fumarate, the consequent hyper-succinylation of several proteins and the inhibition of apoptosis. In line with this data, the reduction of succinylation, obtained by overexpressing the desuccinylase SIRT5 in IDH1-R132C-harboring HT1080 cells, decreases BCL-2 accumulation and slows tumor growth [7,56]. Interestingly, protein succinylation and epigenetic changes are two events strictly interconnected in tumors with high levels of mitochondrial oncometabolites. Histones have about 30% lysine targets of succinylation. Notably, histone and chromatin succinylation have been correlated with an increased transcription of the succinylated gene [34], although the detailed mechanisms of how histone and non-histone protein succinylation affects tumorigenesis are still unexplored [19]. The main effects of oncometabolites on cancer biology are summarized in Figure 1.

## 3. Mitochondrial Oncometabolites and Drug Resistance

The first indirect indication linking mitochondria-derived oncometabolites and drug resistance is that tumors producing high amounts of oncometabolites are characterized by highly aggressive phenotype and poor prognosis. Indeed, SDH deficiency is found in about 5–10% of gastrointestinal stromal tumors, which affect younger patients, are resistant to tyrosine kinase inhibitors and have a higher recurrence rate after surgical resection [14]. A similar phenotype characterizes extra-adrenal paragangliomas with by SHD mutations that have early onset, high aggressiveness and poor prognosis [57]. A poor survival has also been observed in patients affected by KIRP with FH deficiency, where FH levels are positively correlated with overall survival. The poor prognosis in FH deficient tumors is explained by the down-regulation of the anti-metastatic miR-200a and miR-200b, and by the activation of the EMT program [46,48]. In line with these data, the analysis of kidney renal clear cell carcinoma (KIRC) TCGA database, showed that the levels of FH mRNA and protein negatively correlate with vimentin, positively correlate with E-cadherin and patients’ survival, confirming the role of FH loss in tumor malignancy and patient poor outcome [46]. Moreover, fumarate accumulation is linked with endometrial cancer aggressiveness, via adenylosuccinate lyase (ADSL) and killer cell lectin-like receptor C3 (KLRC3): the knock-down of ADSL decreases KLRC3 that in turn reduces cell proliferation, migration and invasion. Fumarate recovers KLRC3 expression in ADSL-knocked down cells, counteracting these anti-tumor effects and contributing to cancer aggressiveness [28]. Accordingly, in GBM, a tumor where oncometabolites are often increased, the fumarate-dependent increase of KLRC3 has been correlated with radio-resistance and poor outcome [58]. FH deficiency also protects cancer cells from drugs targeting mitochondrial ETC and causing a metabolic catastrophe, as demonstrated in FH-deficient UOK262 cells treated with ONC201 [59], an anti-GBM agent that decreases OXPHOS-mediated production of ATP [60].

In contrast with the finding suggesting that fumarate facilitates tumor aggressiveness and resistance, in HeLa cells the increase of fumarate and malate chemosensitizes to cisplatin, as demonstrated by slower cell proliferation and reduced tumor size in mice. However, in this work the increase of fumarate seemed a consequence of the decreased level of adenylate kinase 4, a key enzyme in regulating the high-energy phosphoryl transfer reactions, rather than a deficiency of FH [61]. This discrepancy leads to hypothesize that a TCA-independent increase of fumarate may have opposite effects than a TCA-dependent increase in terms of chemotherapy efficacy.

Succinate is linked to chemoresistance because it stabilizes HIF-1α, a transcription factor with a known role in drug resistance. First, as a consequence of the metabolic shift toward a more glycolytic and acidic TME, HIF-1α inactivates weak bases chemotherapeutic agents as anthracyclines. Moreover, it stimulates angiogenesis and EMT program, and increases several drug efflux transporters, such as P-glycoprotein, multidrug resistance-related protein 1 (MRP1) and breast cancer resistance protein [35]. Considering the plethora of chemotherapeutic drugs effluxed by these transporters, tumors with high levels of HIF-1α stabilized by succinate are characterized by a multidrug resistant phenotype. Lastly, the hyper-succinylation increases also NRF2 that protects tumors from the chemotherapy-induced oxidative damages by upregulating anti-oxidant enzymes, drug-inactivating enzymes and MRP1 [62].

IDH1/IDH2-mutated tumors are also resistant to a plethora of drugs. One of the most promising drugs for the treatment of refractory or relapsed AML is enasidenib/AG-221, a IDH2 inhibitor [60]. However, two mutations of IDH2 in the wild-type allele, specifically Q316E and I319M, induce resistance to enasidenib because they alter the binding site between the drug and the IDH2 dimer. The presence of these mutations in trans with the R140Q gain-of-function mutation on the second allele further enhances the resistance to enasidenib [63]. Some cases of resistance to IDH-targeting therapies, such as enasidenib/AG-221 for IDH2 or ivosidenib/AG-120 for IDH1, are caused by an isoform switch induced by the treatment that shifts the prevailing isoform between the cytoplasmic mutant IDH1 and the mitochondrial mutant IDH2. This switch has been reported in a four-case study, and in chondrosarcoma cells harboring the double mutations IDH1-R132G/IDH2-R172V and IDH1-R132H/IDH2-R172S. In these situations, the selective pressure caused by the treatment with a single IDH inhibitor confers a growth advantage to the subclones with prevalent activity of the IDH isoform not inhibited by the treatment [64]. This process is at the basis of the acquired resistance towards IDH-inhibitors and is common particularly in hematological cancers, where IDH1 and IDH2 mutations often co-exist. The use of the combined IDH1 and IDH2 inhibitors may likely prevent the onset of resistance due to the IDH isoform switch. However, this approach has the risks of undesired toxicity and unexpected drug-drug interaction.

**Figure 2:**
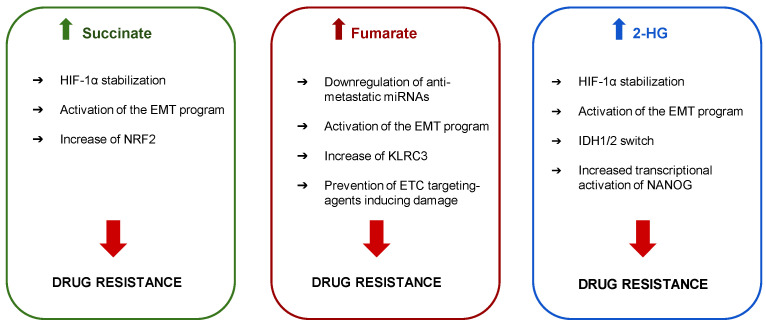
Effects of oncometabolites in drug resistance. The accumulation of oncometabolites such as succinate, fumarate and 2-hydroxyglutarate (2-HG) activates different mechanisms leading to drug resistance such as stabilization of hypoxia inducible factor-1α (HIF-1α), downregulation of anti-metastatic and oncosuppressor miRNAs, induction of epithelial-mesenchymal transition (EMT), overexpression of nuclear factor erythroid 2-related factor 2 (NRF2) and of the killer cell lectin like receptor C3 (KLRC3), prevention of the damages elicited by electron transport chain (ETC)-targeting agents, switch between isocitrate dehydrogenase (IDH) 1 and 2, activation of the stemness regulator NANOG.

IDH mutations confer the resistance also to other anti-cancer agents different from IDH inhibitors. In solid tumors like GBM, IDH1 mutations prevail and confer not only drug resistance but also radioresistance, because of the increased activity of DDR systems and the high activity of de novo and salvage pathways of nucleotide synthesis [65]. GBM cells carrying the R132H-IDH1 are specifically resistant to histone deacetylase inhibitors (HDACi), such as trichostatin A, vorinostat and valproic acid as a consequence of the 2-HG-induced transcriptional increase of NANOG [66], a key regulator of stemness and self-renewal properties of cancer cells. Mechanisms oncometabolites mediated drug resistance are summarized in Figure 2.

## 4. Pharmacological Approaches to Reduce Mitochondrial Oncometabolites

**Figure 3:**
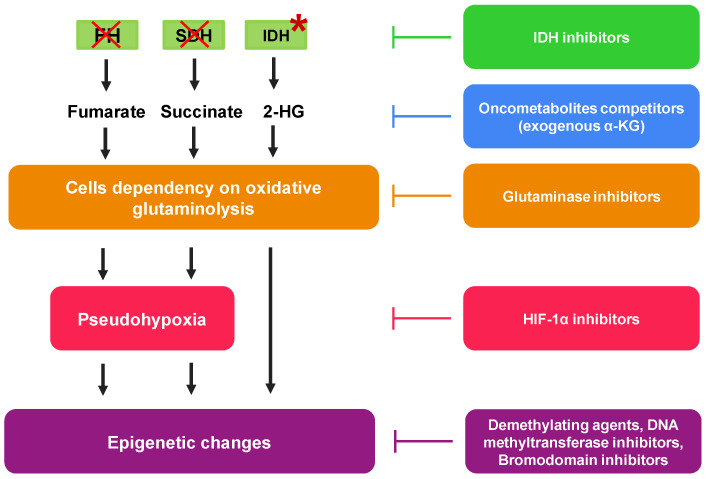
Overview of the main pharmacological strategies to prevent the synthesis and the effects of oncometabolites. Different pharmacological strategies are currently being investigated to counteract the action of oncometabolites. Some of them target the enzyme that produces the oncometabolites, as isocitrate dehydrogenase (IDH) inhibitors, while others target the downstream effects of oncometabolites, such as glutaminolysis, pseudohypoxia or epigenetic changes. Differently, other approaches aim at competing with the oncometabolites themselves, such as the administration of exogenous α-ketoglutarate (α-KG). (*) Gain-of-function mutations in isocitrate dehydrogenase (IDH); (X) loss-of-function mutations in succinate dehydrogenase (SDH) and fumarate hydratase (FH).

On the one hand the metabolic re-arrangements of cancer cells with over-production of TCA-derived oncometabolites increase tumor aggressiveness; on the other hand, these tumors expose some metabolic vulnerabilities, offering new therapeutic opportunities to eradicate the cells over-producing oncometabolites (Table 1; Figure 3).

Since succinate and fumarate accumulation mainly derive from loss-of-function mutations in SDH and FH, a genetic correction reintroducing the deficient gene is still challenging and poorly feasible. Most attempts to counteract succinate and fumarate accumulation target the downstream pathways activated by these oncometabolites. Indeed, as shown in SDHB knocked-out cells, cells with deficient SDH become extremely dependent on the oxidative glutaminolysis to meet their energy requirement. Therein, they are selectively killed by the glutaminase 1 inhibitors compound 968 and CB-839 [37]. Similarly, also the bromodomain proteins inhibitor JQ1 displays a high selectivity against SDH deficient cells, because it down-regulates c-Myc which transcriptionally enhances glutaminolysis enzymes [37]. Targeting glutaminolysis or its controllers is therefore an effective strategy to eradicate succinate hyper-producing cells. Succinate accumulation caused by SDH deficiency inhibits pyruvate dehydrogenase and stabilizes HIF-1α, but these effects are counteracted by exogenous αKG. The latter appeared indeed an effective antitumor and antiangiogenic compound in SHD-deficient tumors [67,68,69]. Moreover, since HIF-1α is activated by succinate, the use of HIF-1α inhibitors has been proposed as an alternative and effective strategy to block the tumor progression driven by succinate [36]. The major limitations to the use of HIF-1α inhibitors in patients are the high side-effect toxicity, due to the inhibition of the physiological functions of HIF-1α, and the limited anti-tumor efficacy, likely because several downstream effectors of HIF-1α become independent drivers of oncogenesis and progression (https://clinicaltrials.gov/ct2/results/details?cond=Cancer&term=HIF), accessed on April 2021.

Differently from succinate and fumarate, the accumulation of 2-HG may be overcome by the inhibition of mutated IDH. The first drug introduced against IDH mutated AML, venetoclax, was not a direct inhibitor of IDH, but it inhibited the anti-apoptotic protein BCL-2, which is increased by 2-HG [33,53]. After venetoclax, several direct inhibitors of IDH have been tested in clinical trials to antagonize 2-HG, but only two of them—enasidenib/AG-221 and ivosidenib/AG-120—have been approved for the treatment of refractory AML [70]. Enasidenib is a small molecule inhibiting mutant IDH2, which is overexpressed in hematological cancers: it has high selectivity, good solubility and oral bioavailability [33]. Ivosidenib, also characterized by good oral bioavailability, shows a preferential inhibition on IDH1 and is evaluated for solid tumors including GBM [33]. Since in some cases the tumors display the shift between IDH1 and IDH2 mutant isoforms, making isoform-selective inhibitors progressively ineffective [64], dual IDH1/IDH2 inhibitors are under evaluation. One of the most promising dual inhibitors is vorasidenib, and has been proposed for the treatment of GBM since it is able to cross the blood brain barrier [71]. Importantly, the response to IDH inhibitors is affected by the type of mutations [72]. Indeed, it has been demonstrated that R132Q mutation alters the catalytic site structure in such a way that the IDH1 inhibitors ML309, AGI-5198 and GSK864 are less potent. By contrast they are instead more active against R132H-IDH1 or wild-type IDH1 expressing cells [72]. This aspect must be carefully considered in choosing the right inhibitor for the right mutant tumor.

Besides specific inhibitors, several miRNA-targeting agents are also considered since it has been clearly demonstrated that miR-181a had inhibitory effect on IDH1 and miR-183 on IDH2 [73,74]. However, despite the promising results obtained in vitro and in preclinical models, miRNA technology is still problematic in clinical use. Therefore, its efficacy in targeting IDH1/IDH2 activated tumors must be re-evaluated when technical issues concerning delivery, stability and specificity will be overcome.

**Table 1:** Main pharmacological approaches counteracting oncometabolites effects on cancer progression and drug resistance.

Targeted Oncometabolite	Mutated Gene	Drugs
Succinate	SDH	Compound 968 and CB-839 [37]
Fumarate	FH	JQ1 [37] Exogenous αKG [67,68,69] HIF-1α inhibitors [36]
2-HG	IDH	Venetoclax [33,53]. Enasidenib/AG-221 [33,70] Ivosidenib/AG-120 [33,64] Vorasidenib [71] ML309 [72] AGI-5198 [72] GSK864 [72] Azacytidine [75,76] Decitabine [75,76] Temozolomide [77,78]

2-HG: 2-hydroxyglutarate; SDH: succinate dehydrogenase; FH: fumarate dehydratase; IDH: isocitrate dehydrogenase; αKG: α-ketoglutarate; HIF-1α: hypoxia-induced transcriptional factor.

Notably, IDH mutant tumors are characterized by hyper-methylation of DNA, as a result of the epigenetic changes induced by TCA-derived oncometabolites [12]. Hence, some demethylating agents are under investigation in IDH mutated tumors. For instance, the DNA methyltransferase inhibitors azacytidine and decitabine have showed clinical benefits in AML, including IDH mutated leukemias [75,76]. Interestingly, IDH1 and IDH2 mutated GBM, SDHB deficient pheochromocytoma and paraganglioma are more sensitive to temozolomide (TMZ), and IDH1/2 mutations are con-sidered a good prognostic factor. This is likely explained by the hypermethylation of the O6-methylguanine DNA methyltransferase promoter, one of the key antagonists of TMZ activity, in mutated tumors [77,78]. Consistently, the exposure of GBM cells to exogenous 2-HG slows down tumor progression and has a synergistic effect with chemotherapeutic agents inhibiting DNA demethylation, such as azacytidine and decitabine, or inducing DNA damage as daunorubicin [79]. This example shows that oncometabolites cannot be considered entirely under a negative light, because high levels of 2-HG may exert anti-tumor and chemosensitizing effects in IDH1/IDH2 mutated GBM.

It remains to be clarified, however, which factor between the level of oncometabolites or the oncogenic/oncosuppressor pathways altered by mutated TCA enzymes play the most prominent role in chemosensization or chemoresistance.

## 5. Conclusions

The involvement of mitochondria metabolism in cancer growth, progression and drug resistance has been established since a long time. However, many processes related to mitochondrial metabolic reprogramming and linked to drug resistance still need to be explored in depth. Among them, great attention has been recently paid to the mechanisms underlying aggressiveness and drug resistance associated with defects of SDH and FH, and hyper-activating mutations of IDH, all leading to the accumulation of mitochondrial oncometabolites. The generation of a pseudohypoxic phenotype, the epigenetic changes and the post-translational modification in specific proteins induced by the oncometabolites have been regarded as the main mechanism promoting cancer progression. The mechanisms behind the drug resistance displayed by tumors with high levels of succinate, fumarate and 2-HG are in part similar. For instance, oncometabolite-induced resistance often relies on the oncometabolite-driven activation of HIF1-α and EMT programs. Oncometabolites also specifically modulate oncogenic and/or oncosuppressor pathways, determining a complex crosstalk between different genetic alterations co-existing in the same tumors, contributing to aggressiveness and resistance.

To slow down tumor progression and improve drugs sensitivity, different therapeutic strategies targeting the mitochondria-derived oncometabolites have been experimented, and some of them reached phase I/II clinical trials. This is the situation of tumors with IDH1/IDH2 mutations that can be treated with small molecules acting as selective inhibitors, with good success in AML and GBM. Since the defects related to SDH and FH are prevalently due to loss of function, the direct targeting of these enzyme with specific activators or with genetic engineering is more difficult. A better strategy, although indirect, is interfering with the pathways activated by succinate and fumarate. Inhibitors of EMT program, which is downstream of all three TCA-derived oncometabolites—may be another approach limiting tumor aggressiveness, invasion and chemoresistance. In this perspective, epigenetic drugs such as demethylating agents and DNA methyltransferase inhibitors that have been demonstrated to block EMT program [80] are of particular interest. Indeed, IDH1 mutants GBM are resistant to HDACi [66]. The combination of the IDH1 inhibitors ivosidenib/AG-120 associated with other epigenetic drugs such as decitabine or azacytabine, may open new therapeutic possibilities for aggressive and chemorefractory tumors producing oncometabolites.

Another intriguing point is understanding the possible cross-talk between each oncometabolite produced within the tumor cell. If defective SDH and FH lead to the accumulation of succinate and fumarate, this derangement of TCA flux can slow down the upstream steps of the cycle. The accumulation of citrate and isocitrate may favor the generation of 2-HG, thus generating tumors with all three oncometabolites increased. Conversely, a hyperactive/mutated IDH subtracts α-KG from the TCA cycle, and this diversion can indirectly reduce the rate of downstream enzymes, thus limiting the production of succinate and fumarate in tumors with wild-type SDH and FH. Of course, the presence of defective forms of SDH and FH produces the accumulation of fumarate and succinate also in case of mutated IDH. Different combination strategies, depending on the relative amounts of 2-HG, fumarate and succinate, and on the activity of their downstream effectors, should be adopted in order to maximize the efficacy of anti-oncometabolite agents.

Since several genetic lesions within a tumor may co-exist, an in-depth genomic profiling of the TCA cycle genes may help identifying the TCA cycle genotypic profile and the best choice of treatment. The presence of different clones within the same tumors, harboring different mutations, further complicates the situation, because different areas within the tumor bulk may behave metabolically differently. To partially circumvent this issue, the coupling of high-throughput metabolomic analysis and high-resolution imaging analysis by MALDI-imaging techniques can map the intratumor areas with different levels of TCA cycle metabolism and identify the most prominent metabolic phenotype within the tumor mass. The simultaneous advancement in diagnostic techniques and oncometabolite-targeting agents will help the development of an “oncometabolite-based precision medicine” in the next future.

Indeed, despite the limited number of drugs targeting the mitochondria-derived oncometabolites currently available, the research in this field can open very interesting therapeutic opportunities. First tumors with mutations in TCA offer the possibility of targeting either the TCA cycle mutated enzymes or the downstream pathways controlled by the oncometabolite, leading the way to different combination therapies that can be exploited with anti-tumor, anti-metastatic or chemosensitizer purpose. Second, it is noteworthy that the agents targeting the production of mitochondrial derived oncometabolite are rather tumor-selective, because they hit isoforms of the TCA cycle enzymes detected in tumors, but not in non-transformed cells. Therefore, the pharmacological development of these drugs is particularly attractive, because it may lead to the realization of the first “metabolic targeted therapy” in the oncological field, conceived as a multi-target therapy peculiarly effective against tumors resistant to conventional treatments.

## Data Availability

The data presented in this study are openly available in https://clinicaltrials.gov/ct2/results/details?cond=Cancer&term=HIF).
